# Supplementary material for: NLRP3-induced systemic inflammation controls the development of JAK2V617F mutant myeloproliferative neoplasms
Source: Nat Commun. 2025 Nov 26;16:10591. doi: 10.1038/s41467-025-65673-4 (PMC12658227; doi:10.1038/s41467-025-65673-4)
Supplement: Supplementary file 1 — Supplementary Information [file 41467_2025_65673_MOESM1_ESM.pdf]

Supplementary Figures

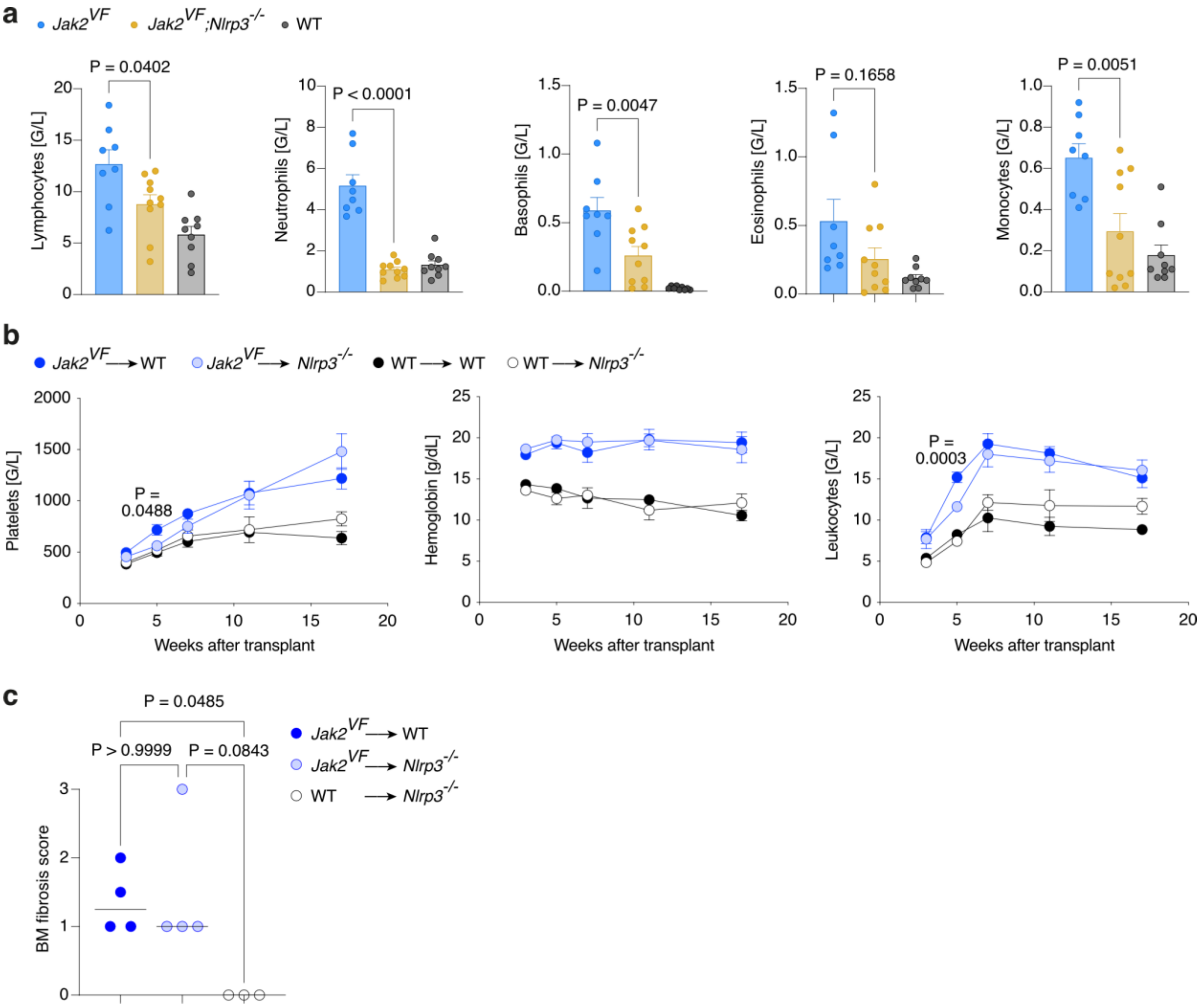

Fig. S1. NLRP3 in radioresistant cells has no sustained effect on blood counts in murine MPN

(a) Differential blood counts of non-transplanted *Jak2*^{VF} (n = 8), *Jak2*^{VF}*;Nlrp3*$^{-/-}$ (n = 10) and WT (n = 9) mice at 25 weeks of age. Blood was collected from the abdominal vena cava. The significant differences between *Jak2*^{VF} and *Jak2*^{VF}*;Nlrp3*$^{-/-}$ are displayed. Scatter bar plots show mean + SEM with dots representing individual mice.

(b) Blood counts of lethally irradiated WT and *Nlrp3*$^{-/-}$ mice transplanted with *Jak2*^{VF} or WT bone marrow. Blood was drawn by submandibular method. For clarity, only the significant differences between *Jak2*^{VF} → WT and *Jak2*^{VF} → *Nlrp3*$^{-/-}$ mice are shown. Plots show mean + SEM (n = 10 mice/group).

(c) Fibrosis of bone marrow was scored from 0 to 3. Dots represent individual mice and horizontal lines the median. *Jak2*^{VF} → WT (n = 4), *Jak2*^{VF} → *Nlrp3*$^{-/-}$ (n = 4) and WT → *Nlrp3*$^{-/-}$ (n = 3).

Statistically significant differences were determined by one-way ANOVA with two-sided Holm-Šidák multiple comparison test (a), mixed-effects model with two-sided Dunnett's multiple comparisons test (b) and Kruskal-Wallis test with two-sided Dunn's multiple comparisons test (c). Source data are provided as a Source Data file.

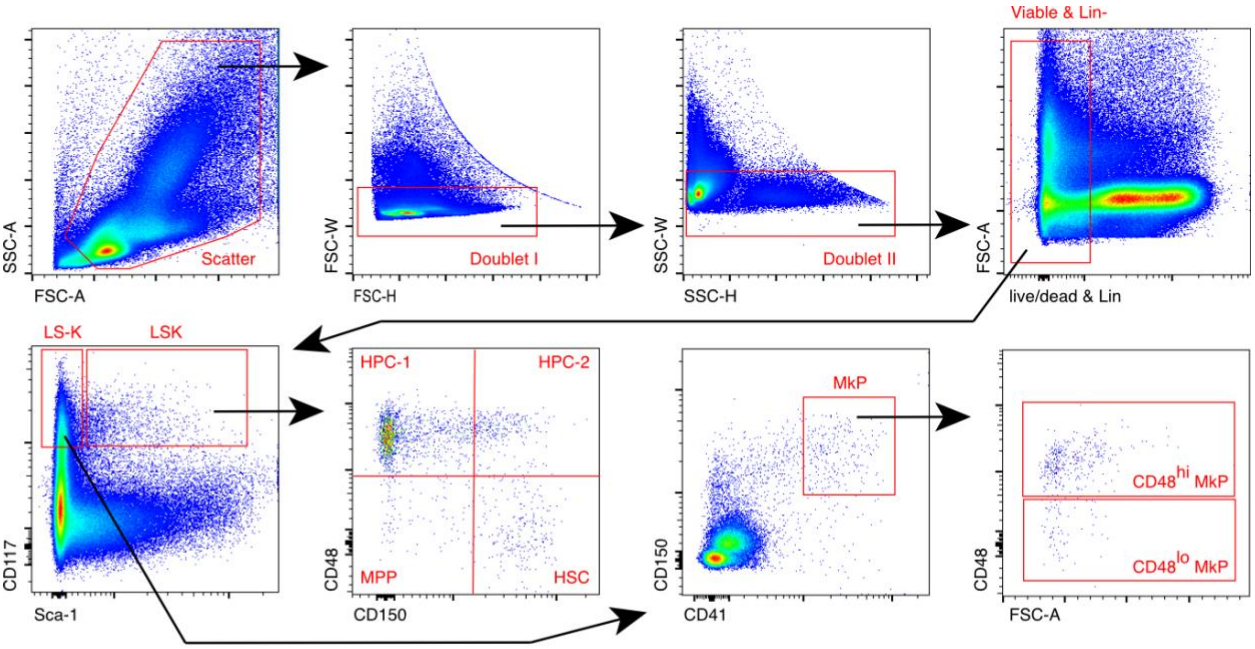

Fig. S2. Flow cytometric identification of bone marrow HSPCs

Representative example of gating for bone marrow HSPCs. LS-K (Lin−, Sca-1−, CD117+), LSK (Lin−, Sca-1+, CD117+), HSC (Lin−, Sca-1+, CD117+, CD150+, CD48−), MPP (Lin−, Sca-1+, CD117+, CD150−, CD48−), HPC-1 (Lin−, Sca-1+, CD117+, CD150−, CD48+), HPC-2 (Lin−, Sca-1+, CD117+, CD150+, CD48+), MkP (Lin−, Sca-1−, CD117+, CD150+, CD41+).

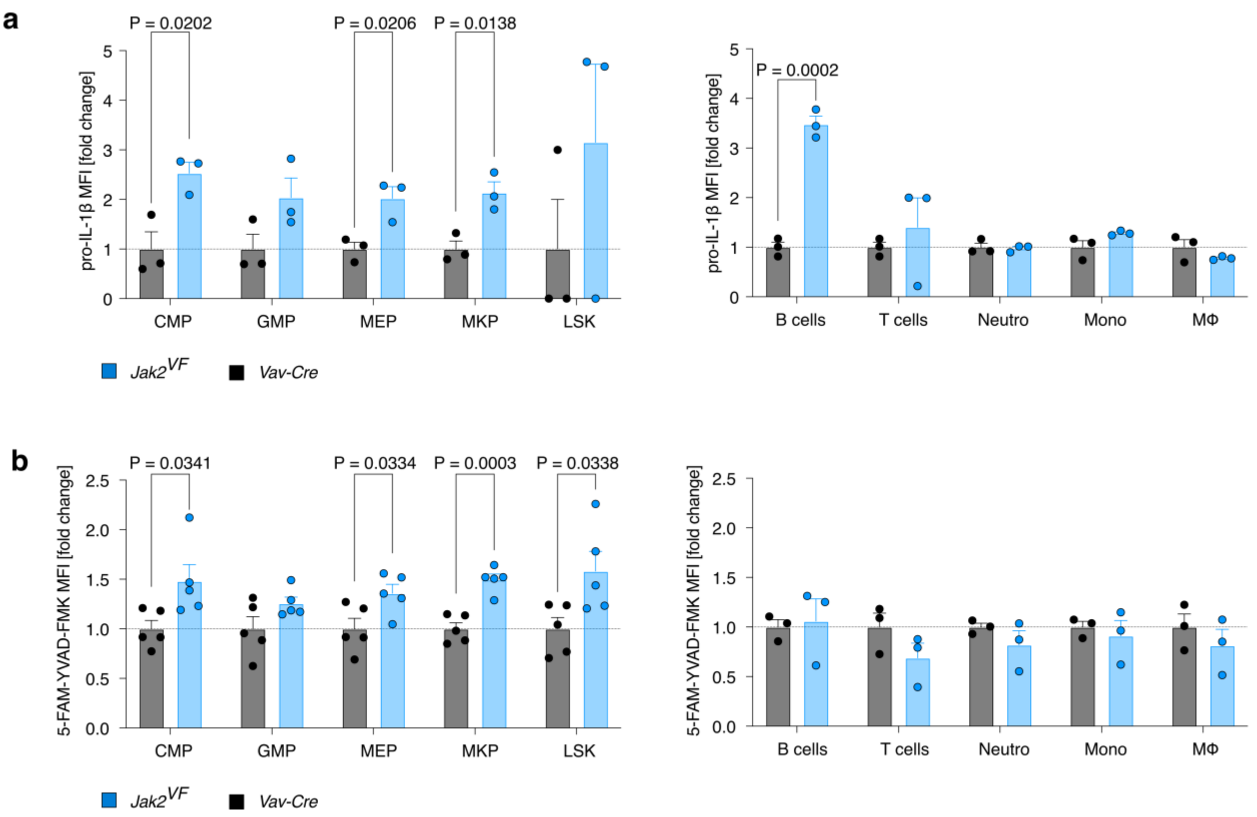

Fig. S3. Cell-type specific inflammasome activation

(a) Fold change of pro-IL-1β mean fluorescence intensity (MFI) of indicated cell types from *Jak2*^{VF} and *Vav-Cre* mice (n = 3 mice/group).

(b) Fold change of 5-FAM-YVAD-FMK mean fluorescence intensity (MFI) of indicated cell types from *Jak2*^{VF} and *Vav-Cre* mice. HSPC subsets (left, n = 5 mice/group) and mature immune cells (right, n = 3 mice/group).

Plots show mean + SEM. Statistically significant differences were determined by two-tailed unpaired t-test. Source data are provided as a Source Data file.

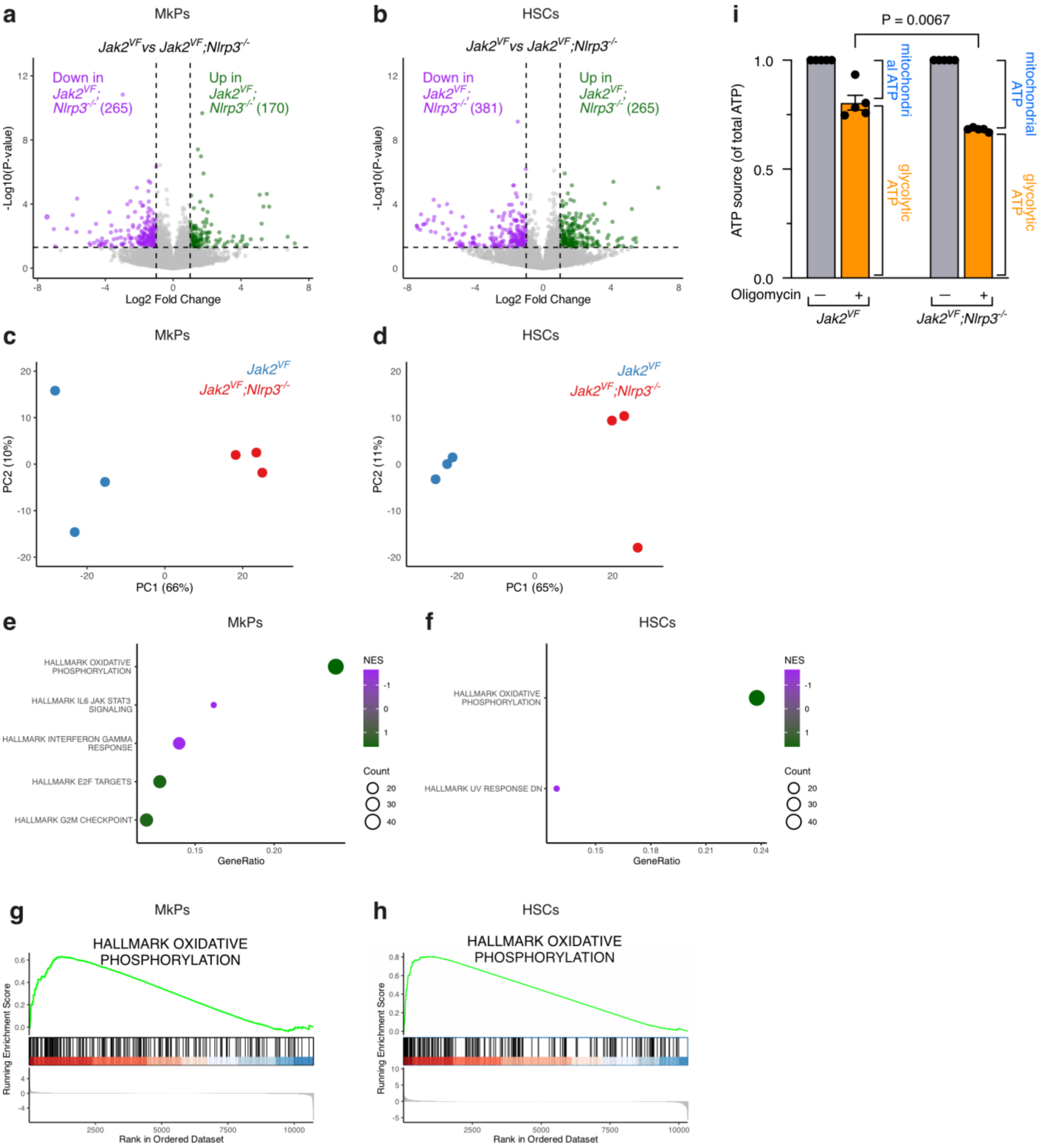

Fig. S4. Deleting *Nlrp3* shifts HSPC metabolism towards OXPHOS.

Global gene expression analysis (a-h) by 3' -mRNA seq of bone marrow MkPs and HSCs from *Jak2*^{VF} and *Jak2*^{VF};*Nlrp3*$^{-/-}$ mice (n = 3 mice/group).

(a-b) Volcano plots indicating transcriptomic changes. Genes with a non-adjusted p value < 0.05 and a signed fold change > 2 are colored.

(c-d) PCA plots based on differentially expressed genes (non-adjusted p value < 0.05).

(e-f) Dot plots of GSEA results for Hallmark gene sets significantly up- or downregulated in *Jak2*^{VF}*;Nlrp3*$^{-/-}$ compared to *Jak2*^{VF} mice (adjusted p value < 0.05).

(g-h) GSEA plots for the Hallmark oxidative phosphorylation gene set.

(i) Measurement of ATP levels with or without the OXPHOS inhibitor oligomycin in bone marrow cells from *Jak2*^{VF} and *Jak2*^{VF}*;Nlrp3*$^{-/-}$ mice (n = 5 mice/group) to calculate the relative amount of ATP synthesized by mitochondria, Plots show mean + SEM. Statistically significant differences were determined by two-tailed unpaired t-test. Source data (i) are provided as a Source Data file.

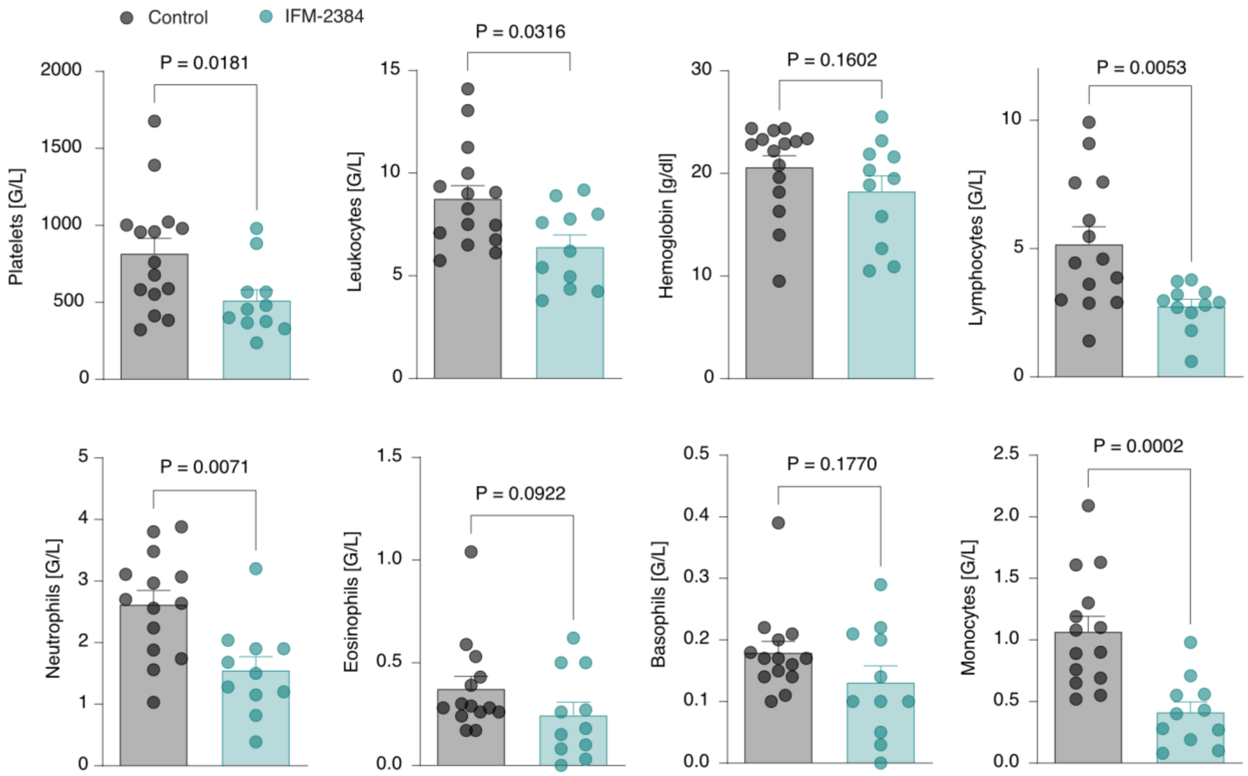

Fig. S5. NLRP3 inhibition decreases platelets and leukocytes, including neutrophils, in *Jak2^{VF}* BM mice.

Differential blood counts of *Jak2^{VF}* BM mice fed IFM-2384 (n = 11) or control chow (n = 15) 20 weeks after treatment start. Blood was collected from the abdominal vena cava.

Scatter bar plots show mean + SEM with dots representing individual mice. Statistically significant differences were determined by two-tailed unpaired Mann-Whitney U test. Source data are provided as a Source Data file.
