# Supplementary material for: NLRP3-induced systemic inflammation controls the development of JAK2V617F mutant myeloproliferative neoplasms
Source: Nat Commun. 2025 Nov 26;16:10591. doi: 10.1038/s41467-025-65673-4 (PMC12658227; doi:10.1038/s41467-025-65673-4)
Supplement: Supplementary file 2 — Reporting Summary [file 41467_2025_65673_MOESM2_ESM.pdf]

nature portfolio

Corresponding author(s): Lino L. Teichmann

Last updated by author(s): Aug 13, 2025

Reporting Summary

Nature Portfolio wishes to improve the reproducibility of the work that we publish. This form provides structure for consistency and transparency in reporting. For further information on Nature Portfolio policies, see our Editorial Policies and the Editorial Policy Checklist.

Statistics

For all statistical analyses, confirm that the following items are present in the figure legend, table legend, main text, or Methods section.

n/a	Confirmed	
☐	☒	The exact sample size (*n*) for each experimental group/condition, given as a discrete number and unit of measurement
☐	☒	A statement on whether measurements were taken from distinct samples or whether the same sample was measured repeatedly
☐	☒	The statistical test(s) used AND whether they are one- or two-sided<br>*Only common tests should be described solely by name; describe more complex techniques in the Methods section.*
☒	☐	A description of all covariates tested
☐	☒	A description of any assumptions or corrections, such as tests of normality and adjustment for multiple comparisons
☐	☒	A full description of the statistical parameters including central tendency (e.g. means) or other basic estimates (e.g. regression coefficient) AND variation (e.g. standard deviation) or associated estimates of uncertainty (e.g. confidence intervals)
☐	☒	For null hypothesis testing, the test statistic (e.g. *F*, *t*, *r*) with confidence intervals, effect sizes, degrees of freedom and *P* value noted<br>*Give P values as exact values whenever suitable.*
☒	☐	For Bayesian analysis, information on the choice of priors and Markov chain Monte Carlo settings
☒	☐	For hierarchical and complex designs, identification of the appropriate level for tests and full reporting of outcomes
☐	☒	Estimates of effect sizes (e.g. Cohen's *d*, Pearson's *r*), indicating how they were calculated

Our web collection on statistics for biologists contains articles on many of the points above.

Software and code

Policy information about availability of computer code

Data collection	Flow cytometry - Canto II (BD Biosciences) or Northern Lights (Cytek)<br>Cells sorting - FACSAria II (BD Biosciences)<br>RNA-seq - NovaSeq 6000 System (Illumina)<br>qRT-PCR - RealPlex 2 Thermocycler (Eppendorf)<br>Multiplex bead-based assay - FlexMAP 3D System (Luminex)
Data analysis	Flow cytometry - FlowJo 10.8.1 (BD)<br>RNA-seq - Lexogen's web-based Kangooroo platform, R (4.4.2), DESeq2 (1.46.0), clusterProfiler (4.14.6), ggplot2 (3.5.1), enrichplot (1.26.6)<br>Statistical analysis - GraphPad 9 (Prism)<br>Clustering analysis and visualization - R (4.4.2), ComplexHeatmap (2.14.0)

For manuscripts utilizing custom algorithms or software that are central to the research but not yet described in published literature, software must be made available to editors and reviewers. We strongly encourage code deposition in a community repository (e.g. GitHub). See the Nature Portfolio guidelines for submitting code & software for further information.

Data

Policy information about availability of data

All manuscripts must include a data availability statement. This statement should provide the following information, where applicable:

- Accession codes, unique identifiers, or web links for publicly available datasets
- A description of any restrictions on data availability
- For clinical datasets or third party data, please ensure that the statement adheres to our policy

3'-mRNA sequencing data have been deposited in the Zenodo repository and can be accessed via https://doi.org/10.5281/zenodo.16893730.

Research involving human participants, their data, or biological material

Policy information about studies with human participants or human data. See also policy information about sex, gender (identity/presentation), and sexual orientation and race, ethnicity and racism.

Reporting on sex and gender	Sex and gender were not considered in this study.
Reporting on race, ethnicity, or other socially relevant groupings	Race, ethnicity, or other socially relevant categorizations were not used in this study.
Population characteristics	173 MPN patients from the German Study Group MPN (GSG-MPN) bioregistry and 37 healthy controls. No specific population characteristics were considered as covariates in this study.
Recruitment	MPN patients were recruited through the outpatient clinics where they receive treatment. Healthy controls were recruited at various outpatient clinics of the University Hospital Bonn. In both cases, participation was initiated by the treating physician, minimizing the potential for self-selection bias.
Ethics oversight	MPN blood samples were obtained from patients enrolled in the German Study Group Myeloproliferative Neoplasms (GSG-MPN) bioregistry, approved by the Institutional Review Boards of the participating centers (DRKS-ID: DRKS00006035). Healthy control blood samples were drawn at the University Hospital of Bonn and collection was approved by the Ethics Committee of the Medical Faculty of the University of Bonn (154/13).

Note that full information on the approval of the study protocol must also be provided in the manuscript.

Field-specific reporting

Please select the one below that is the best fit for your research. If you are not sure, read the appropriate sections before making your selection.

[x] Life sciences [ ] Behavioural & social sciences [ ] Ecological, evolutionary & environmental sciences

For a reference copy of the document with all sections, see nature.com/documents/nr-reporting-summary-flat.pdf

Life sciences study design

All studies must disclose on these points even when the disclosure is negative.

Sample size	As a preclinical study, no sample-size calculation was performed. Instead, sample sizes were determined based on previous experience and sample sizes employed in similar studies to obtain reproducibility and statistical significance. The chosen sample sizes exceed the minimum threshold required to achieve adequate power in standard statistical tests for detecting moderate effect sizes. The number of animals was optimized to balance scientific rigor with the 3R principles.
Data exclusions	No data were excluded.
Replication	All animal experiments were repeated at least twice. All attempts at replication were successful. Independent experiments were pooled and analyzed together whenever possible.
Randomization	For pharmacological inhibition studies mice were allocated randomly to control or experimental group. In all other mouse studies, random allocation was not applicable, as mice were assigned to groups based on genotype. In these cases, all comparisons were made between genetically defined groups. In studies involving human samples, comparison were made between MPN patients and healthy controls.
Blinding	The investigators were blinded to group allocation during data collection and analysis.

Reporting for specific materials, systems and methods

We require information from authors about some types of materials, experimental systems and methods used in many studies. Here, indicate whether each material, system or method listed is relevant to your study. If you are not sure if a list item applies to your research, read the appropriate section before selecting a response.

Materials & experimental systems

n/a	Involved in the study	
[ ]	[x]	Antibodies
[ ]	[x]	Eukaryotic cell lines
[x]	[ ]	Palaeontology and archaeology
[ ]	[x]	Animals and other organisms
[ ]	[x]	Clinical data
[x]	[ ]	Dual use research of concern
[x]	[ ]	Plants

Methods

n/a	Involved in the study	
[x]	[ ]	ChIP-seq
[ ]	[x]	Flow cytometry
[x]	[ ]	MRI-based neuroimaging

Antibodies

Antibodies used

PE-conjugated anti-ASC (clone HASC-71, Cat.# 653904, mouse antibody, 1:100 dilution, Biolegend),
PerCP/Cyanine5.5-conjugated anti-CD3 (clone 17A2, Cat.# 100218, rat antibody, 1:200 dilution, Biolegend),
Pacific Blue-conjugated anti-CD11b (clone M1/70, Cat.# 101224, rat antibody, 1:200 dilution, Biolegend),
Pacific Blue-conjugated anti-CD11c (clone N418, Cat.# 117322, Armenian hamster antibody, 1:200 dilution, Biolegend),
PerCP/Cyanin 5.5-conjugated anti-CD19 (clone 6D5, Cat.# 115532, rat antibody, 1:200 dilution, Biolegend),
FITC-conjugated anti-CD34 (clone RAM34, Cat.# 11-0341-82, rat antibody, 1:100 dilution, Invitrogen),
Pacific Blue-conjugated anti-CD41 (clone MWReg30, Cat.# 133932, rat antibody, 1:200 dilution, Biolegend),
PE-conjugated anti-CD42b (clone Xia.G5, Cat.# M040-2, rat-antibody, dilution 1:40, Emfret),
Brilliant Violet 510-conjugated anti-CD48 (clone HM48-1, Cat.# 103443, Armenian hamster antibody, 1:200 dilution, Biolegend),
APC-conjugated anti-CD117 (clone ACK2, Cat.# 135108, rat antibody, 1:100 dilution, Biolegend),
PE-conjugated anti-CD150 (clone TC15-12F12.2, Cat.# 115904, rat antibody, 1:200 dilution, Biolegend),
APC-conjugated anti-CD201 (EPCR) (clone RCR-16, Cat.# 351906, rat antibody, 1:200 dilution, Biolegend),
Alexa Fluor 488-conjugated anti-GbVI (clone FAB6758G, Cat.# FAB6758G, rat antibody, 1:100 dilution, R&D),
Pacific Blue-conjugated anti-F4/80 (clone BM8, Cat.# 123124, rat antibody, 1:400 dilution, Biolegend),
PerCP-conjugated anti-Gr-1 (clone RB6-8C5, Cat.# 108426, rat antibody, 1:200 dilution, Biolegend),
FITC-conjugated anti-ICAM (clone YN1/1.7.4., Cat.# 116105, rat antibody, 1:100 dilution, Biolegend),
FITC-conjugated anti-Ki-67 (clone 16A8, Cat.# 652410, rat antibody, 1:400 dilution, Biolegend),
APC-conjugated anti-Ly6C (clone HK1.4, Cat.# 128016, rat antibody, 1:800 dilution, Biolegend),
PerCP-conjugated anti-Ly6G (clone 1A8, Cat.# 127654, rat antibody, 1:200 dilution, Biolegend),
APC-conjugated anti-NLRP3 (clone REA668, Cat.# 130-111-210, human antibody 1:100 dilution, Milteny),
FITC-conjugated anti-pro-IL-1β (clone NJTEN3, Cat.# 11-7114-82, rat antibody, 1:100 dilution, Invitrogen),
FITC-conjugated rat IgG1κ isotype control antibody (clone RTK2071, Cat.# 400406, rat antibody, 1:100 dilution, Biolegend; used as control antibody in the pro-IL-1β staining),
PE-Cy7-conjugated anti-Sca-1 (clone D7, Cat.# 25-5981-82, rat antibody, 1:300 dilution, Invitrogen),
PerCP-conjugated, anti-Ter-119 (clone Ter119, Cat.# 116226, rat antibody, 1:200 dilution, Biolegend),
Unconjugated anti-CD16/32 (Trustain, clone 93, Cat.# 101320, rat antibody, 1:400 dilution, Biolegend),
PE-conjugated anti-VCAM (clone M/K-2, Cat.# RMCD10604, rat antibody, 1:40 dilution, Invitrogen)

Validation

PE-conjugated anti-ASC (clone HASC-71, Cat.# 653904, mouse antibody, 1:100 dilution, Biolegend) was validated by the vendor: https://www.biolegend.com/de-de/sean-tuckers-tests/pe-anti-asc-tms-1-antibody-10627,
PerCP/Cyanine5.5-conjugated anti-CD3 (clone 17A2, Cat.# 100218, rat antibody, 1:200 dilution, Biolegend) was validated by the vendor: https://www.biolegend.com/de-de/products/percp-cyanine5-5-anti-mouse-cd3-antibody-5596,
Pacific Blue-conjugated anti-CD11b (clone M1/70, Cat.# 101224, rat antibody, 1:200 dilution, Biolegend) was validated by the vendor: https://www.biolegend.com/de-de/products/pacific-blue-anti-mouse-human-cd11b-antibody-3863,
Pacific Blue-conjugated anti-CD11c (clone N418, Cat.# 117322, Armenian hamster antibody, 1:200 dilution, Biolegend) was validated by the vendor: https://www.biolegend.com/de-de/products/pacific-blue-anti-mouse-cd11c-antibody-3864,
PerCP/Cyanin 5.5-conjugated anti-CD19 (clone 6D5, Cat.# 115532, rat antibody, 1:200 dilution, Biolegend) was validated by the vendor: https://www.biolegend.com/de-de/products/percp-anti-mouse-cd19-antibody-4260,
FITC-conjugated anti-CD34 (clone RAM34, Cat.# 11-0341-82, rat antibody, 1:100 dilution, Invitrogen) was validated by the vendor: https://www.thermofisher.com/antibody/product/CD34-Antibody-clone-RAM34-Monoclonal/11-0341-82,
Pacific Blue-conjugated anti-CD41 (clone MWReg30, Cat.# 133932, rat antibody, 1:200 dilution, Biolegend) was validated by the vendor: https://www.biolegend.com/de-de/products/pacific-blue-anti-mouse-cd41-antibody-16505,
PE-conjugated anti-CD42b (clone Xia.G5, Cat.# M040-2, rat-antibody, dilution 1:40, Emfret) was validated by the vendor: https://www.emfret.com/index.php?id=shop&no_cache=1&tx_feproducts_pi1%5Ba%5D=details&tx_feproducts_pi1%5BCatNo%5D=M0402&cHash=2236b51dbc0bd2e5a38aa40bfc3f149b,
Brilliant Violet 510-conjugated anti-CD48 (clone HM48-1, Cat.# 103443, Armenian hamster antibody, 1:200 dilution, Biolegend) was validated by the vendor: https://www.biolegend.com/de-de/products/brilliant-violet-510-anti-mouse-cd48-antibody-14066,
APC-conjugated anti-CD117 (clone ACK2, Cat.# 135108, rat antibody, 1:100 dilution, Biolegend) was validated by the vendor: https://www.biolegend.com/de-de/products/apc-anti-mouse-cd117-c-kit-antibody-6358,
PE-conjugated anti-CD150 (clone TC15-12F12.2, Cat.# 115904, rat antibody, 1:200 dilution, Biolegend) was validated by the vendor: https://www.biolegend.com/de-de/products/pe-anti-mouse-cd150-slam-antibody-1369?GroupID=BLG10572,
APC-conjugated anti-CD201 (EPCR) (clone RCR-16, Cat.# 351906, rat antibody, 1:200 dilution, Biolegend) was validated by the vendor: https://www.biolegend.com/de-de/products/apc-anti-human-cd201-epcr-antibody-7693,
Alexa Fluor 488-conjugated anti-GbVI (clone FAB6758G, Cat.# FAB6758G, rat antibody, 1:100 dilution, R&D) was validated by the

vendor: https://www.rndsystems.com/products/mouse-gpvi-alexa-fluor-488-conjugated-antibody-784808_fab6758g,
Pacific Blue-conjugated anti-F4/80 (clone BM8, Cat.# 123124, rat antibody, 1:400 dilution, Biolegend) was validated by the vendor: https://www.biolegend.com/de-de/products/pacific-blue-anti-mouse-f4-80-antibody-4075,
PerCP-conjugated anti-Gr-1 (clone RB6-8C5, Cat.# 108426, rat antibody, 1:200 dilution, Biolegend) was validated by the vendor: https://www.biolegend.com/de-de/products/percp-anti-mouse-ly-6g-ly-6c-gr-1-antibody-4287,
FITC-conjugated anti-ICAM (clone YN1/1.7.4., Cat.# 116105, rat antibody, 1:100 dilution, Biolegend) was validated by the vendor: https://www.biolegend.com/de-de/products/fitc-anti-mouse-cd54-antibody-1679,
FITC-conjugated anti-Ki-67 (clone 16A8, Cat.# 652410, rat antibody, 1:400 dilution, Biolegend) was validated by the vendor: https://www.biolegend.com/de-de/products/fitc-anti-mouse-ki-67-antibody-8573,
APC-conjugated anti-Ly6C (clone HK1.4, Cat.# 128016, rat antibody, 1:800 dilution, Biolegend) was validated by the vendor: https://www.biolegend.com/de-de/products/apc-anti-mouse-ly-6c-antibody-6047,
PerCP-conjugated anti-Ly6G (clone 1A8, Cat.# 127654, rat antibody, 1:200 dilution, Biolegend) was validated by the vendor: https://www.biolegend.com/de-de/products/percp-anti-mouse-ly-6g-antibody-13351,
APC-conjugated anti-NLRP3 (clone REA668, Cat.# 130-111-210, human antibody 1:100 dilution, Milteny) was validated by the vendor: https://www.miltenyibiotec.com/DE-en/products/nlrp3-nalp3-antibody-anti-human-mouse-reafinity-rea668.html#conjugate=apc:size=100-tests-in-200-ul,
FITC-conjugated anti-pro-IL-1β (clone NJTEN3, Cat.# 11-7114-82, rat antibody, 1:100 dilution, Invitrogen) was validated by the vendor: https://www.thermofisher.com/antibody/product/IL-1-beta-Pro-form-Antibody-clone-NJTEN3-Monoclonal/11-7114-82,
FITC-conjugated rat IgG1κ isotype control antibody (clone RTK2071, Cat.# 400406, rat antibody, 1:100 dilution, Biolegend; used as control antibody in the pro-IL-1β staining) was validated by the vendor: https://www.biolegend.com/en-us/products/fitc-rat-igg1-kappa-isotype-ctrl-1828,
PE-Cy7-conjugated anti-Sca-1 (clone D7, Cat.# 25-5981-82, rat antibody, 1:300 dilution, Invitrogen) was validated by the vendor: https://www.thermofisher.com/antibody/product/Ly-6A-E-Sca-1-Antibody-clone-D7-Monoclonal/25-5981-82,
PerCP-conjugated, anti-Ter-119 (clone Ter119, Cat.# 116226, rat antibody, 1:200 dilution, Biolegend), was validated by the vendor: https://www.biolegend.com/de-de/products/percp-anti-mouse-ter-119-erythroid-cells-antibody-4291,
Unconjugated anti-CD16/32 (Trustain, clone 93, Cat.# 101320, rat antibody, 1:400 dilution, Biolegend) was validated by the vendor: https://www.biolegend.com/de-at/clone-search/trustain-fcx-anti-mouse-cd16-32-antibody-5683?GroupID=BLG9237,
PE-conjugated anti-VCAM (clone M/K-2, Cat.# RMCD10604, rat antibody, 1:40 dilution, Invitrogen) was validated by the vendor: https://www.thermofisher.com/antibody/product/VCAM-1-Antibody-clone-M-K-2-Monoclonal/RMCD10604

Eukaryotic cell lines

Policy information about cell lines and Sex and Gender in Research

Cell line source(s)	32D cells (DSMZ, ACC 411) were provided by Prof. Steffen Koschmieder (Department of Hematology, Oncology, Hemostaseology, and Stem Cell Transplantation, Faculty of Medicine, RWTH Aachen University, Aachen, Germany)
Authentication	32D cells were only authenticated by their morphology.
Mycoplasma contamination	32D cells tested negative for mycoplasma contamination
Commonly misidentified lines (See ICLAC register)	32D cells used in this study are not listed in the ICLAC database of commonly misidentified cell lines.

Animals and other research organisms

Policy information about studies involving animals; ARRIVE guidelines recommended for reporting animal research, and Sex and Gender in Research

Laboratory animals	Vav-Cre;Jak2V617F/+ C57BL/6 mice (Jak2VF) were generated and kindly provided by Jean-Luc Villeval (Hasan et al. Blood, 2013). Jak2VF;Nlrp3-/- mice were generated by interbreeding Jak2VF with Nlrp3-/- C57BL/6 mice (Millennium Pharmaceuticals). C57BL/6J mice were purchased from Jackson Laboratories. Mice were maintained under special pathogen free (SPF) conditions, housed in individually ventilated cages (IVCs) in groups of no more than five animals. The housing rooms were standardized to a constant air temperature of approximately 22°C, a humidity level of 50–60%, and up to 15 air changes per hour. The day-night cycle was centrally controlled and consisted of 12 hours of light and 12 hours of darkness. The age at which the mice were sacrificed is specified in the manuscript.
Wild animals	NA
Reporting on sex	In bone marrow transplantation experiments, 10–12-week-old recipients were injected intravenously with bone marrow cells harvested from 6–8-week-old donors. In all groups, care was taken to balance the ratio of female to male donors. Female mice were used as recipients. In all other experiments, female and male mice were used in close to equal numbers.
Field-collected samples	NA
Ethics oversight	Experimental procedures were performed in accordance with the German Animal Welfare Act and approved by the State Agency for Nature, Environment and Consumer Protection, NRW.

Note that full information on the approval of the study protocol must also be provided in the manuscript.

Clinical data

Policy information about clinical studies
All manuscripts should comply with the ICMJE guidelines for publication of clinical research and a completed CONSORT checklist must be included with all submissions.

Clinical trial registration: DRKS-ID: DRKS00006035

Study protocol: https://drks.de/search/en/trial/DRKS00006035/details

Data collection: The GSG-MPN-Registry is a non-interventional prospective study with longitudinal patient recruitment (different time points for patients to enter the registry). All patients who meet the WHO criteria for BCR-ABL1-negative MPN, who are at least 18 years of age, and have given written informed consent can be included.

Outcomes:
Primary outcome:
- Registration of patients with BCR-ABL1-negative myeloid neoplasms
- Preservation of bone marrow, peripheral blood, and if necessary, urine samples , germline material in the biomaterial banks in Aachen and Ulm
- Documentation of clinical characteristics and epidemiological data at diagnosis and in Follow up, using a defined catalog of variables
- Evaluation of prognostic and predictive markers
- Documentation of the most important patient-relevant clinical endpoints: overall survival (OS) and quality of life at diagnosis and in Follow up

Secondary outcome:
- Documentation of further patient-relevant clinical endpoints (outcomes): response rates, disease-associated mortality, transformation rate
- Documentation and evaluation of the quality of therapy and diagnosis using quality indicators
- Validation of published prognostic factors within the registry cohort and search for new possible prognostic factors
- Correlation of clinical endpoints with chosen therapy
- Collection and description of new therapies/ supportive care

Plants

Seed stocks: NA

Novel plant genotypes: NA

Authentication: NA

Flow Cytometry

Plots

Confirm that:

- [x] The axis labels state the marker and fluorochrome used (e.g. CD4-FITC).
- [x] The axis scales are clearly visible. Include numbers along axes only for bottom left plot of group (a 'group' is an analysis of identical markers).
- [x] All plots are contour plots with outliers or pseudocolor plots.
- [x] A numerical value for number of cells or percentage (with statistics) is provided.

Methodology

Sample preparation: To prepare bone marrow cells from mice, femurs were extracted and soft tissue removed with a scalpel and by gentle rolling over paper towels. Femurs were cut open on one side and placed into a 0.5 ml tube with a small hole at the tip, which was inserted into another 1.5 ml tube. Bone marrow cells were flushed from the bone by centrifugation. For splenocytes, spleens were removed, minced and pressed through a metal strainer. All cells were treated with Red Blood Cell Lysis buffer (Biolegend) and suspensions were filtered through 70 µm Nitex nylon mesh before further use.
For human PBMCs, whole blood was collected into EDTA tubes, and PBMCs were isolated by density gradient centrifugation with lymphocyte separation medium (PromoCell). PBMCs were either immediately used or cryopreserved at -80°C in RPMI1640 complemented with 20% FCS and 10% DMSO. Monocytes were isolated from PBMCs using the Pan Monocyte Isolation Kit (Milteny).

Instrument	Canto II (BD Biosciences) or Northern Lights (Cytek)<br>FACSAria II (BD Biosciences)
Software	FlowJo 10.8.1 (BD)
Cell population abundance	Cell subsets were sorted with >98% purity as as controlled by by remeasurement of of sorted populations.
Gating strategy	Cells were initially gated by by FSC/SSC, followed by by viability dye to to exclude dead cells. Further detail gating strategies were provided in in supplementary figures.

☒ Tick this box to confirm that a figure exemplifying the gating strategy is provided in the Supplementary Information.
